# Emotional reasoning and anxiety sensitivity: Associations with social anxiety disorder in childhood^☆^

**DOI:** 10.1016/j.jad.2013.09.014

**Published:** 2014-01

**Authors:** Anna Alkozei, Peter J. Cooper, Cathy Creswell

**Affiliations:** a Winnicott Research Unit, School of Psychology and Clinical Language Sciences, University of Reading, Reading RG6 6AL, UK; b Department of Psychology, Stellenbosch University, South Africa

**Keywords:** Child, Anxiety, Cognition, Social anxiety disorder, Emotional reasoning, Anxiety sensitivity

## Abstract

**Background:**

Two specific cognitive constructs that have been implicated in the development and maintenance of anxiety symptoms are anxiety sensitivity and emotional reasoning, both of which relate to the experience and meaning of physical symptoms of arousal or anxiety. The interpretation of physical symptoms has been particularly implicated in theories of social anxiety disorder, where internal physical symptoms are hypothesized to influence the individual's appraisals of the self as a social object.

**Method:**

The current study compared 75 children on measures of anxiety sensitivity and emotional reasoning: 25 with social anxiety disorder, 25 with other anxiety disorders, and 25 nonanxious children (aged 7–12 years).

**Results:**

Children with social anxiety disorder reported higher levels of anxiety sensitivity and were more likely than both other groups to view ambiguous situations as anxiety provoking, whether physical information was present or not. There were no group differences in the extent to which physical information altered children's interpretation of hypothetical scenarios.

**Limitations:**

This study is the first to investigate emotional reasoning in clinically anxious children and therefore replication is needed. In addition, those in both anxious groups commonly had comorbid conditions and, consequently, specific conclusions about social anxiety disorder need to be treated with caution.

**Conclusion:**

The findings highlight cognitive characteristics that may be particularly pertinent in the context of social anxiety disorder in childhood and which may be potential targets for treatment. Furthermore, the findings suggest that strategies to modify these particular cognitive constructs may not be necessary in treatments of some other childhood anxiety disorders.

## 1 Introduction

Anxiety disorders are common in childhood, are associated with high levels of emotional distress, and result in academic and social impairment (Essau et al., 2002; Ezpeleta et al., 2001; Mychailyszyn et al., 2010).One of the most common anxiety disorders in childhood is social anxiety disorder, affecting up to 7% of young people (Chavira et al., 2004). It is characterized by an intense and irrational fear of embarrassment in social situations. Cognitive Behavior Therapy (CBT) has been shown to be an effective treatment for childhood anxiety disorders, however around 40% of children continue to experience significant levels of anxiety following such treatment (Cartwright-Hatton et al., 2004; In-Albon and Schneider, 2007). Recent findings suggest that children with social anxiety disorder may do less well from generic anxiety treatments than those with other anxiety disorders (Ginsburg et al., 2011; Hudson et al., 2010), suggesting that the processes that maintain social and other anxiety disorders may differ. In order to improve the treatment outcomes of anxious children, particularly those with social anxiety, a better understanding of these processes is required.

The importance of cognitions in the development and maintenance of anxiety disorders has been emphasized for both adults (Beck et al, 1985) and children (Kendall, 1985). Central to these models is the hypothesis that interpretation biases (e.g., overestimation of danger in the environment) drive avoidant behaviors and anxious affect, which then reinforce those same cognitions. Furthermore, models of social anxiety disorder place as central the tendency of socially anxious individuals to display interpretation biases regarding their internal physical symptoms and to use these symptoms to form a negative impression of themselves as a social object (e.g., “If my hands shake/I blush/or show other signs of anxiety, people will think I am incompetent/odd/stupid”), which then leads to behaviors, and cognitive and somatic symptoms that maintain social anxiety (Clark and Wells, 1995).

There is extensive empirical support for an interpretation bias towards external threat among anxious children: clinically anxious children and adolescents, as well as highly anxious children from community samples (7–14 years), are more likely than nonanxious controls to interpret ambiguous stories in a threatening manner (Barrett et al., 1996; Chorpita et al., 1996a; Creswell et al., 2005; Muris et al., 2000a, b). When samples have been restricted to pre-adolescent children however, studies have often failed to find significant differences in threat interpretation between children with anxiety disorders and non-anxious children (i.e., 8–12 years; Waters et al., 2008; 7–12 years, Creswell et al., 2013), although, notably, there is recent evidence that anxious children as young as 4 years may show an interpretation bias towards threat in comparison to their nonanxious counterparts (Dodd et al., 2012). In addition, one study showed that group differences in response to ambiguous stories became more apparent with increasing age (Creswell et al., 2013). It is well known that children's cognitive development changes throughout childhood (Piaget, 1970). Whereas younger children's cognitive style is marked by greater egocentricism, with maturity children learn to view situations from a range of different perspectives. Ambiguous scenarios require children to consider a situation from a variety of different standpoints. It is therefore possible that while younger children with anxiety disorders do not differ from nonanxious children in their threat interpretations, with increasing age children develop the cognitive ability to consider a variety of different explanations for ambiguous scenarios and therefore features that are associated with children's anxiety disorders become more apparent.

In addition to interpretation biases in response to external stressors, two specific interpretation biases regarding internal symptoms have been implicated in the maintenance of anxiety disorders, namely ‘anxiety sensitivity’ and ‘emotional reasoning’. Both of these constructs relate to the experience and personal significance of physical symptoms and to arousal or anxiety.

Anxiety sensitivity (AS) is defined as the belief that the experience of bodily symptoms related to anxiety has negative implications in terms of causing illness, loss of control (e.g., feeling sick might entail medical consequences), embarrassment (e.g., feeling shaky is regarded as visible to others), or additional anxiety (e.g., a fast heart rate is the cause for concern; Reiss and McNally, 1985). There is evidence that AS is more marked among clinically anxious and highly anxious community children (9–14 years) in comparison to non-anxious children (Muris et al., 2001; Muris, 2002; Rabian et al., 1993; Vasey et al., 1995). Whereas anxiety sensitivity is most strongly associated with panic disorder in adulthood (Olatunji and Wolitzky-Taylor, 2009), panic disorder is rare in childhood (Ollendick et al., 1994) and, in fact, Essau et al. (2010) showed, in a large community sample (*N*=1292) of adolescents (12–17 years), that AS was most strongly associated with symptoms of social anxiety (*r*=.50) in comparison to all other anxiety disorder symptoms, including panic symptoms (*r*=.40). The age range investigated however, makes it difficult to draw conclusions about AS and social anxiety in childhood. In addition, as some items relate to embarrassment associated with symptoms of anxiety, which is central to models of social anxiety disorder, it is unclear whether these findings are the result of socially anxious children scoring higher specifically on items related to embarrassment, or whether they hold more general negative beliefs about anxiety-related bodily symptoms.

A construct related to AS is emotional reasoning (ER) which refers to the tendency to draw conclusions about a situation, on the basis of one's own bodily experience rather than objective information (e.g., Arntz et al., 1995; Muris et al., 2003b). Unlike in adulthood, where differences in ER between anxious and nonanxious populations have been found (Arntz et al., 1995), studies with children have shown that there is a general tendency for all children (aged 7–14 years) to interpret stories as more threatening when the character in the story is experiencing a physical reaction than when information about physical symptoms is omitted. This effect is particularly pronounced among highly anxious children (Muris et al., 2003a, b; Morren et al., 2004). Muris et al. (2008) suggested that ER may change with age and experience, as children learn that the occurrence of physical symptoms might have a range of different meanings other than fear or anxiety. Specifically, it may be that differences between anxious and nonanxious children are less apparent at a younger age, but that with increasing age, nonanxious children may show a reduced tendency to rate stories that include physical information as threatening whereas anxious children may not show this developmental change. Whether this interaction between age and anxiety disorder status is associated with ER in childhood has yet to be directly tested. Furthermore, all studies of ER in childhood reported to date have been limited to community populations and replication with clinical samples is needed. Given the tentative support for greater AS among socially anxious adolescents, and in view of the importance of physical symptoms in models of the maintenance of social anxiety disorder (Clark and Wells, 1995), an association between social anxiety disorder and greater ER might well obtain.

In summary, there is some suggestion that there may be elevated levels of AS and ER amongst anxious children, however this remains to be tested in relation to social anxiety disorder. Furthermore, the potential moderating impact of age needs to be elucidated. We set out to address this gap in the literature by examining ER and AS in clinically anxious and non-anxious children. In order to ensure that findings were not accounted for by common comorbid conditions, we took into account symptoms of low mood and behavioral disturbance. The specific hypotheses tested were as follows:

1. Children with a current anxiety disorder will display greater AS and ER in comparison to non-anxious children.
2. Children with current social anxiety disorder will display greater AS and ER than both non-anxious children and children with other forms of anxiety (who do not have social anxiety disorder).
3. Differences in the level of AS and ER between children with anxiety disorders and non-anxious children will become more pronounced with increasing age.

## 2 Method

### 2.1 Participants

Seventy five children aged 7–12 years took part in the study. 25 children met diagnostic criteria for social anxiety disorder (SA), 25 children met diagnostic criteria for an anxiety disorder but not social anxiety disorder (ANX), and 25 were children selected on the basis of having anxiety levels within a non-clinical range (NONANX). There were equal numbers of male (*N*=10) and female (*N*=15) participants in each group, and groups did not differ significantly on mean age (months) (*F*(2, 74)=.05, *p*=.95). Non-anxious control participants were volunteers, recruited through invitation letters sent through schools, specifically asking for children to form a non-anxious comparison group. The inclusion criteria were that children must be within 7–12 years and have anxiety levels within the normal range, based on both parent and child report (SCAS-c/p; see below).

Table 1 illustrates children's anxiety and comorbid diagnoses for the SA and the ANX group. Children in the SA group had a significantly greater number of diagnoses compared to children in the ANX condition (*t*(48)=3.78, *p*<.001). This can be explained by children in the SA group meeting criteria for social anxiety disorder (mean number of diagnoses for the SA group, *M*=3.10, mean number of diagnoses for the ANX group, *M*=1.80). Children did not differ significantly on meeting criteria for any other diagnosis (see Table 1). Rates of mood and behavioral disorder were slightly elevated in the SA group, although the groups did not differ significantly on the prevalence of either disorder (*χ²*(1)=2.0, *p*=.16 and *χ²*(1)=2.23, *p*=.33 respectively).

Analyses were conducted to confirm group differences on total and social anxiety symptoms. As expected, significant group differences were found on total anxiety (SCAS-c, *F*(2, 68)=28.83, *p*<.001, *d*=1.16; SCAS-p, *F*(2, 64)=50.88, *p*<.001, *d*=2.37) and social anxiety symptom scores (SCAS-c social anxiety, *F*(2, 68)=22.75, *p*<.001, *d*=1.35; SCAS-p social anxiety, *F*(2, 64)=24.39, *p*<.001, *d*=1.27). As shown in Table 2, post-hoc tests identified significant differences between both clinical groups and the control group for total SCAS scores. For the social anxiety subscale, there was a significant difference between the SA group and both other groups, as well as a significant difference between the ANX group and the NONANX group. When scores for social anxiety were subtracted from total anxiety scores, differences between the three groups remained significant (SCAS-c, *F*(2, 57)=24.68, *p*<.001, *d*=1.66; SCAS-p, *F*(2, 57)=58.611, *p*<.001, *d*=2.43), reflecting significant differences between both clinical groups and the control group. There was no difference between the SA and the ANX groups, reflecting the fact that that the higher total SCAS scores found among the SA group was a function of higher levels of social anxiety but not higher levels of other anxiety symptoms.

### 2.2 Measures

*Structured diagnostic interview with children and parents.* Children were assigned diagnoses on the basis of the Anxiety Disorders Interview Schedule for DSM IV for Children-Child and Parent Versions (ADIS-C/P; Silverman and Albano, 1996). Where children met symptom criteria for a diagnosis (based on either child or parent report) they were assigned a clinical severity rating (CSR) ranging from 0 (complete absence of psychopathology) to 8 (severe psychopathology). Only those children who met symptom criteria with a CSR of 4 or more (moderate psychopathology) were considered to meet diagnostic criteria. Assessors (psychology graduates) were trained on the standard administration and scoring of the ADIS-C/P through verbal instruction, listening to assessment audio-recordings and participating in diagnostic consensus discussions. The first twenty ADIS-child and ADIS-parent interviews conducted were then discussed with a consensus team, led by a Consultant Clinical Psychologist. The assessor and the consensus team independently allocated diagnoses and CSRs. Following the administration of 20 child and 20 parent interviews, inter-rater reliability for each assessor was checked, and if assessors achieved reliability of at least.85 they were then required to discuss just one in six interviews with the consensus team to prevent inter-rater drift. Overall reliability for the team was excellent. Reliability for presence or absence of diagnosis was kappa=.98; and for the CSR intra-class correlation=.99.

*Symptoms of anxiety.* The Spence Children's Anxiety Scale (SCAS-c/p; Spence, 1998; Nuata et al., 2004) was administered to assess child and parent reported symptoms of anxiety. Both the child and parent versions require the respondent to rate how often the child experiences each of 38 anxiety symptoms, on a 4 point scale from ‘never’(0) to ‘always’ (3). The SCAS-c/p has demonstrated high internal reliability and concurrent validity (Spence, 1998; Nuata et al., 2004) with children from 7 years of age (Muris et al., 2000c). In addition, the social phobia scale has been found to correlate highly with the Social Anxiety Scale for Children (SASC-R) (Muris et al., 2000b). Internal consistency was good (*α*=.94 for SCAS-c, *α*=.91 for SCAS-p).

*Anxiety sensitivity.* The Childhood Anxiety Sensitivity Index (CASI; Silverman et al., 1991) was administered to assess child self-reported beliefs that physical symptoms are responsible for provoking embarrassment, additional anxiety and illness/loss of control. The scale requires children to rate to what extent they agree with each of 18 statements on a 3 point scale; ‘not at all’ (0), ‘a little bit’ (1) or ‘a lot’ (2). Statements include “It scares me when my heart beats fast”, and “When I’m afraid I worry that I might be going crazy”. It has been shown that the CASI has good test-retest reliability, good item-total correlations, and that it explains significant variance in the prediction of trait anxiety over and above other measures of anxiety in children as young as seven years (Silverman et al., 1991; Chorpita et al., 1996b). In order to examine whether any group differences relate specifically to social items, individual subscale scores were calculated by adding scores for items that aimed to investigate children’s thoughts on the possible negative consequences of physical symptoms in terms of embarrassment (5 items), additional anxiety (9 items) and illness (4 items). Muris et al., (2001) conducted a confirmatory factor analysis that revealed that (most of) these items loaded onto these three different factors. Internal consistency was high for CASI total scores *α*=.93, as well as for CASI additional anxiety (CASI_ADD), *α*=.89, CASI embarrassment (CASI_EMB), *α*=.74, and CASI illness subscale scores (CASI_ILL) *α*=.81.

*Emotional reasoning.* In order to assess whether children used bodily symptoms to validate their fear, Muris et al., (2003b) Emotional Reasoning Stories (ERS) were translated from Dutch into English. The stories described three scenarios that children may encounter and there were two versions of each story: (i) an ambiguous story (AMB), and (ii) an ambiguous story with physical information (AMB+P). The stories described daily situations that children may encounter, but where the presence of threat was unclear and the ending remained relatively ambiguous (e.g., It is your first day at a new school. You do not know any of the children yet who are in your class. The teacher tells you that all students will soon be part of a drama play. While saying that he looks at you. He says, “That sounds like something for you! You should definitely be part of the play!”).

The AMB+P was ident**i**cal to the AMB apart from one additional sentence that was included after the first sentence in each story, relating to a physical symptom the child was experiencing (e.g., ‘You can feel your heart beating’). To avoid order effects, the type of story was alternated. Each story comprised 5 segments containing 1–2 sentences each. The stories were read out sentence by sentence by a research assistant and children were asked after each segment whether they thought the story would have a scary ending or not. Children were interviewed individually by a research assistant as not all children would be expected to be able to read and write confidently and to prevent children from reading the full story before responding rather than responding to questions on the basis of each sentence. If children indicated that the story would end in a scary way, they were asked to rate from zero to 10 how scary the end would be. Finally, the whole story was read out again without interruption and children were asked two open questions: (i) how they would feel if this happened to them, and, (ii) how they thought the story would end. For each story four scores were obtained: (i) fear^1^ threshold – i.e. how quickly (after hearing how many segments) children indicated that they thought that a story would end in a scary way (internal reliability was *α*=.63 for AMB, *α*=.57 for AMB+P); (ii) fear ratings – i.e. the child's prediction of how scary the story would be after each segment (internal reliability was *α*=.91 for AMB, *α*=.88 for AMB+P); (iii) negative feelings – i.e. children's free responses on how they would feel if this situation happened to them; and (iv) threat interpretation – i.e. children's free responses on how they thought the story would end. Scores on each index were combined for each type of story separately (i.e., AMB and AMB+P). Children's free responses regarding their feelings and their threat interpretations were coded by a psychology doctoral student blind to child group and scores on all other measures. Free responses regarding the child's feelings were coded as ‘Negative’ (e.g., ‘I would feel scared’) or ‘Not negative’ (e.g., ‘I would feel happy/normal’). Free responses about children's threat interpretation were coded as ‘Threat’ (‘Everyone will laugh at me.’) or ‘Non-threat’ (‘Nothing will happen.’). A second independent coder (a graduate psychologist) coded a sample of the responses (*n*=20) in order to assess inter-rater reliability. Intraclass correlations were good (for threat interpretations ICC=.90, for negative feelings ICC=.90).

*Ethical considerations.* This study was reviewed by the Local Research Ethics Committee on behalf of the National Health Service and the University of Reading Research Ethics Committee. Parents and children were both provided with written and verbal information about the study. In order to participate in the study written parental and child consent and were both required.

### 2.3 Procedure

Following referral by a health or education service professional to the Berkshire Child Anxiety Clinic at the University of Reading, potential participants were invited for an initial assessment appointment in which the ADIS-C/P and SCAS-c/p were administered to parents and children in an interview format. Where children met criteria for either the SA or ANX groups, they were invited to take part in a laboratory based research assessment before initiating treatment.

On arrival for the lab assessment, children and their parents were introduced to the overall plan for the session, and were given some time to acclimatize. Children were then taken to a neighboring room and were administered the CASI and ERS by the research assistant in an interview format. The child was then taken back to the lab where a range of other tasks were conducted as part of ongoing research in the Berkshire Child Anxiety Clinic.

For the control children, schools were mailed information about the study and asked to send out information sheets, consent forms, and the SCAS-p to all parents of children aged 7–12 years. Parents were asked to read the information sheet and send back the completed consent form and SCAS-p if they were willing for their child to take part. If parents reported on the SCAS-p that children scored within normal range, children were seen individually in their school, informed about the study, and their consent was obtained. Children were then administered the SCAS-c, CASI and ERS in an interview format. Children who scored above normal limits on the SCAS-c were excluded from the current study.

### 2.4 Overview of analytic strategy

Continuous data were screened in relation to the assumptions of parametric testing (Tabachnick and Fidell, 2000). In most cases, variables met the necessary assumptions; however, where assumptions were violated, data were either transformed, confirmatory analyses were conducted on the original data by running analyses with 1000 bootstrap samples, or alternative tests were used as indicated. Where results were rerun with 1000 bootstrap samples, results remained consistent throughout, so only original analyses are reported. As our hypotheses included consideration of group x age interactions, age (in months) was centered using the mean as a reference value to overcome colinearity between the group and group x age variables (unadjusted, *r*(75)=.92, *p*<.001; centered, *r*(75)=−.01, *p*=.93) (Kraemer, 2006).

As the SA group had elevated levels of mood and behavioral disorders (see Table 1), analyses were rerun excluding the 19 cases (13 children in the SA and 6 children in the ANX group) who met criteria for either one of these disorders Results remained consistent throughout.

### 2.5 Anxiety sensitivity

AS was investigated by conducting a univariate analysis of covariance (ANCOVA) with CASI total scores as the dependent variable and group (SA vs ANX vs NONANX) as the independent variable. In addition, a multivariate analysis of covariance (MANCOVA) was conducted with each of the CASI subscales (embarrassment, illness, additional anxiety) as the dependent variables and group as the independent variable. For both analyses, age and the age *x* group interaction were entered as the covariates.

### 2.6 Emotional reasoning

ER was investigated in two steps. We first investigated whether children differed in responses on each type of stories separately (AMB and AMB+P) by conducting two MANCOVAS with fear threshold, fear ratings, threat interpretation and negative feelings as the dependent variables and group as the independent variable. Age and the age x group interaction were entered as covariates.

To examine associations between group and ER, a MANCOVA was conducted with difference scores between AMB+P and AMB as the dependent variables. This approach was taken as a repeated measures multivariate analysis of covariance was not available as a bootstrapped test, nor as a robust test (Field, Miles, & Field, 2012), and the distribution of the difference scores adhered better to a normal distribution than the raw scores. Group was the independent variable, age and the age x group interaction were entered as covariates. As analyses of difference scores cannot establish whether there is an overall ER effect for the whole group regardless of anxiety diagnosis, a paired t-test was conducted first to establish whether the addition of physical information was associated with different responses to the stories.

## 3 Results

### 3.1 Anxiety sensitivity

For total CASI scores there was a significant main effect of group (*F*(2, 69)= 23.71, *p*<.001, *d*=1.18), but no significant effect of age (*F*(1, 69)=2.27, *p*=.14, *d*=.36) or group *x* age interaction (*F*(2, 69)=1.60, *p*=.21, *d*=.30). As shown in Table 3, planned contrasts revealed that the SA group scored significantly higher on the CASI than the ANX group (*k*=9.71, *p*<.001) and the NONANX group (*k*=13.1, *p*<.001), but there was no significant difference between the ANX and NONANX group (*k*=3.34, *p*=.09).

In addition, the overall MANCOVA for the CASI subscales (CASI_EMB, CASI_ADD, CASI_ILL) revealed a significant main effect of group (*F*(3, 68)=15.90, *p*<.001) and a main effect of age (*F*(3, 67)=4.12, *p*<.01), but no significant group x age interaction (*F*(3, 68)=1.15, *p*=.36). Between subjects tests showed that there were significant differences between the three groups on the subscales of embarrassment (*F*(2, 69)=11.71, *p*<.001, *d*=.88), additional anxiety (*F*(2, 69)=22.88, *p*<.001, *d*=1.18), and illness (*F*(2, 69)=15.92, *p*<.001, *d*=.81). As shown in Table 3, planned contrasts revealed that the SA group significantly differed from both other groups on all measures, but that there were no differences between the ANX and NONANX group. Socially anxious children scored higher on embarrassment scores (*k*=1.99, *p*<.01 and *k*=2.97, *p*<.001 respectively), additional anxiety scores (*k*=5.47, *p*<.001 and *k*=7.59, *p*<.001 respectively), and illness scores (*k*=2.24, *p*<.001 and *k*=2.52, *p*<.001 respectively).

**Fig. 1:**
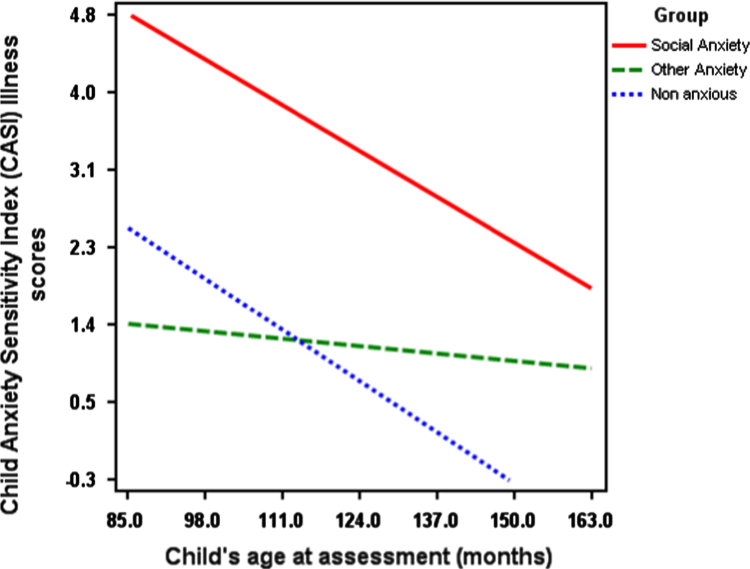
Associations between age, group and Child Anxiety Sensitivity Index (CASI) Illness score.

In addition, between subjects tests revealed that age was significantly associated with illness scores (*F*(1, 69)=8.91, *p*<.01, *d*=.61). Fig. 1 shows the association with age and CASI_ILL scores which illustrates that with increasing age, children's CASI_ILL scores decreased.

### 3.2 Emotional reasoning

For AMB stories, there were significant effects of group (*F*(4, 67)=18.64, *p*<.001) and age (*F*(4, 66)=7.36, *p*<.001), but no significant group x age interaction (*F*(4, 67)=1.12, *p*=.35).

Between subjects tests revealed that there were significant group differences on fear threshold (*F*(2, 69)=16.53, *p*<.001, *d*=.68), fear ratings (*F*(2, 69)=24.11, *p*<.001, *d*=.79), negative feelings (*F*(2, 69)=8.67, *p*<.001, *d*=.75) and threat interpretations (*F*(2, 69)=12.33, *p*<.001, *d*=.78). As shown in Table 4, children in the SA group scored lower than both the ANX and the NONANX groups on scores for fear threshold (*k*=4.25, *p*<.001, and *k*=4.11, *p*<.001 respectively), and higher than the ANX and NONANX groups for fear ratings (*k*=42.88, *p*<.001, and *k*=40.56, *p*<.001 respectively), negative feelings (*k*=.53, *p*<.001, and *k*=.76, *p*<.001 respectively), and threat interpretation (*k*=1.0, *p*<.001, and *k*=1.01, *p*<.001 respectively).

**Fig. 2:**
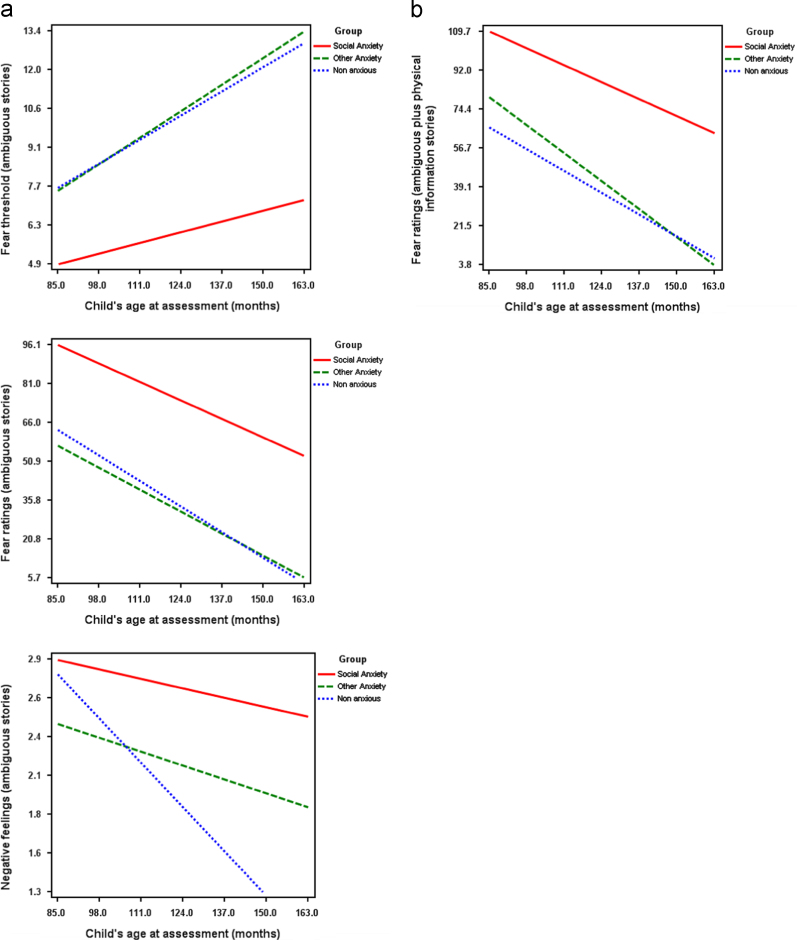
Associations between age (months), group and responses to ambiguous stories. (a) Without physical information. Fear threshold. Fear ratings. Negative feelings. (b) With physical information. Fear ratings.

In addition, age was significantly associated with fear threshold (*F*(1, 69)=11.42, *p*<.001, *d*=.65), fear ratings (*F*(1, 69)=22.16, *p*<.001, *d*=.80) and negative feelings (*F*(1, 69)=10.11, *p*<.001, *d*=.55). It can be seen from Fig. 2a that children in the SA group scored consistently higher (lower for fear threshold) than both other groups, but that with increasing age, fear perception indices decreased for all groups.

For AMB+P stories, there were significant effects of group (*F*(4, 66)=10.68, *p*<.001) and age (*F*(4, 67)=5.32, *p*<.001) but no significant group x age interaction (*F*(4, 67)=1.43, *p*=.22). Between subjects tests indicated that there were significant differences for fear threshold (*F*(2, 69)=14.00, *p*<.001, *d*=.77) and fear ratings (*F*(2, 69)=20.76, *p*<.001, *d*=.95) and negative feelings (*F*(1, 69)=10.11, *p*<.001, *d*=.65). Children in the SA group scored higher than children in the ANX and in the NONANX group. As shown in Table 4, children in the SA group scored lower on fear threshold (*k*=4.05, *p*<.001 and *k*=4.55, *p*<.001 respectively), and higher on fear ratings (*k*=43.55, *p*<.001 and *k*=49.60, *p*<.001 respectively). Age was associated with fear ratings (*F*(1, 69)=21.08, *p*<.001, *d*=.90). It can be seen from Fig. 2b that children in the SA group scored consistently higher on fear ratings than both other groups, but with increasing age, scores for all three groups decreased.

**Fig. 3:**
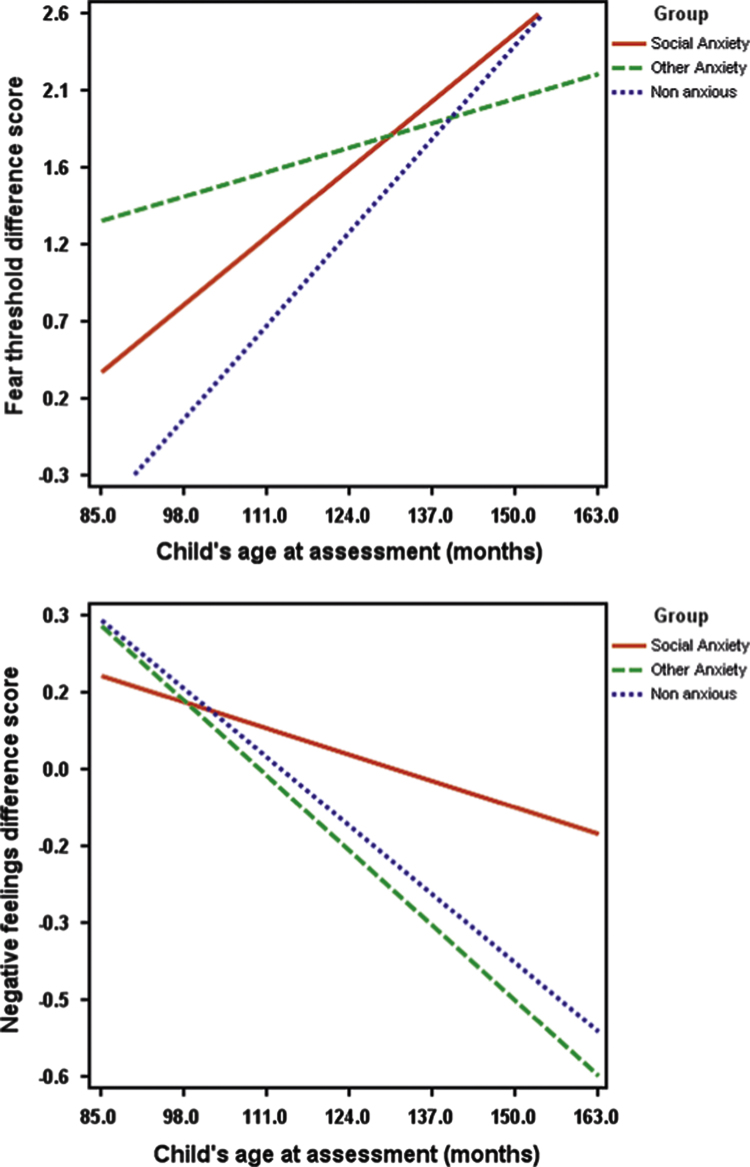
Associations between age (months), group and difference scores between ambiguous plus physical information stories and ambiguous stories without physical information. Fear threshold difference score. Negative feelings difference score.

In comparison to when stories did not include physical information, when stories did include physical information, all children had lower scores for fear threshold (*t*(74)=4.61, *p*<.001, *d*=.54) and higher scores for fear ratings (*t*(74)=4.55, *p*<.001, *d*=.55) and threat interpretation (*t*(74)=1.91, *p*=.06, *d*=.23). With regards to difference scores for fear threshold, fear ratings, negative feelings and threat interpretations (i.e., the difference between responses to stories with and without physical information), there was not a significant effect of group (*F*(4, 67)=1.82, *p*=.13), but there was for age (*F*(4, 66)=5.46, *p*<.001). There was not a significant group x age interaction (*F*(4, 67)=1.48, *p*=.22). It can be seen from Fig. 3 that, with increasing age, children showed greater fear threshold difference scores (*F*(1, 69)=3.80, *p*=.05, *d*=.40) and lower negative feelings difference scores (*F*(1, 69)=6.6, *p*<.05, *d*=.65).

Finally, in order to explore whether AS was associated with ER, the correlation between the CASI total score and difference scores for fear threshold, fear ratings, negative feelings and threat interpretations was analyzed using four separate bivariate correlations. In all cases anxiety sensitivity was not associated with greater emotional reasoning (fear ratings, *r*(73)=.19, *p=*.09; fear threshold, *r*(73)=.18, *p*=.12; negative feelings, *r*(73)=−.07, *p*=.50; threat interpretations, *r*(73)=−.08, *p*=.45).

## 4 Discussion

The aims of this study were to investigate anxiety sensitivity (AS) and emotional reasoning (ER) among children with social anxiety disorder (SA), other anxiety disorders (ANX), and non-anxious children (NONANX). Greater levels of AS were, in most cases, not associated with greater levels of ER, supporting the assumption that AS and ER are separate constructs. The current findings suggest that children with social anxiety disorder may associate negative consequences with physical symptoms; however there was no evidence that the experience of physical symptoms leads them to change their interpretation of ambiguous situations.

In line with our hypotheses, we found greater AS in the SA group compared to both other groups, which corresponds to previous findings in community populations of adolescents (Essau et al., 2010); and this is consistent with models of the maintenance of social anxiety disorder which specify that the belief that anxiety symptoms are likely to have negative consequences is central to social anxiety (e.g., Clark and Wells, 1995). Of note, the findings presented here suggest that children with anxiety disorders other than social anxiety disorder are not more likely to believe that the physical symptoms accompanying anxiety cause embarrassment, illness or additional anxiety compared to nonanxious children. It is possible that associations between anxiety sensitivity and other types of anxiety symptoms found in previous studies were driven by comorbidity with social anxiety symptoms. Essau et al. (2010), for example, did not take into account the potential influence of comorbid social anxiety symptoms when investigating associations between different anxiety symptom subscales (i.e. separation anxiety symptoms, generalized anxiety symptoms, panic symptoms) and anxiety sensitivity. Indeed, the findings from this study suggest that there may be an association between social anxiety disorder, specifically, and anxiety sensitivity in children. This is consistent with the hypothesis that negative beliefs about physical symptoms maintain social anxiety disorder by promoting avoidance of socially challenging situations in which children may experience anxiety-related physical symptoms. It needs to be acknowledged, however, that a recent study with a community twin population did not find that social anxiety symptoms are specifically associated with greater anxiety sensitivity (e.g. Waszczuk et al., 2013), and therefore further work in this area is needed. In addition, experimental studies are now required to evaluate whether children's appraisals of internal physical information influence their affect and behavior in stressful situations (and whether this is specific to social situations, as proposed by Clark and Wells, 1995), as well as treatment studies to determine whether modifying socially anxious children's cognitions about their physical symptoms might improve treatment outcomes.

With regards to ER, our findings correspond in part with those of Muris et al. (2003a, b) who showed that children (8–12 years), regardless of their level of anxiety symptoms, showed a general tendency to rate stories as more threatening when physical information was included. The absence of differences between groups in terms of ER in our study, however, is contrary to findings from previous studies. Muris et al. (2003b), for example, found an emotional reasoning effect among highly anxious children although this was only in relation to ratings of negative feelings. This discrepancy might be the result of differences in the measurement of anxiety (i.e., objective diagnostic assessments versus self-report symptom measures), the study populations (clinic versus community), and the variation in culture and language (Dutch versus British). Our results require replication, however they suggest that emotional reasoning does not discriminate clinically anxious and non-anxious children, and that differences between clinical and non-clinical groups may emerge at later stages in development.

With regards to age effects, the extent to which children viewed anxiety-related physical symptoms as indicating illness decreased as age increased. This is consistent with the suggestion that children are able to consider a broader range of explanations for physical symptoms as they get older (Muris et al., 2008). Similarly, with increasing age children's reports on indices of fear and threat in response to ambiguous scenarios reduced, and the addition of physical symptoms led to lower thresholds to decide a story was anxiety provoking and greater fear ratings compared to when no physical symptoms were included in the scenarios.

Contrary to our hypotheses, differences between groups did not become significantly more pronounced on either anxiety sensitivity or emotional reasoning with increasing child age. Notably, one recent study did find that group differences in children's cognitive biases emerged with increasing age (Creswell et al., 2013), however the age by group interaction was specifically found for a measure of perceived control (and not threat interpretation or anticipated negative emotions). These findings highlight the importance of pinpointing the type of cognitive content that distinguishes anxious and nonanxious children at different ages. Further investigations including a broader range of cognitive indices and a broader age range, including adolescents, would be of value to examine whether group differences begin to emerge later in childhood or adolescence.

Finally, it is striking that children with social anxiety disorder scored consistently higher on fear and threat indices than both other-anxious and non-anxious children which is in partial contrast to a recent study that failed to find such differences (Creswell et al., 2013). It may be that participant characteristics (e.g., the current study had less male participants (40% males in the present study versus 47% males in the previous study) and more frequent comorbid mood disorders (40% in the present study versus 25% in the previous study)) account for this discrepancy. In addition in the current study the assessment materials included a more limited range of scenarios (3), whereas previous studies have used a broader range of social and nonsocial scenarios (12) (e.g., Barrett et al., 1996; Creswell et al., 2005, 2013). One possibility is that the contexts in which social anxiety is associated with threat-related interpretation biases are fairly specific and group differences are diluted when a broad range of scenarios are presented. These differences across studies highlight the need to clearly identify the particular contexts (e.g., types of scenarios) in which highly anxious children, in particular those with social anxiety disorder, show cognitive distortions. Consistent with previous studies (Creswell et al., 2013; Waters et al., 2008), however, children with anxiety disorders other than social anxiety disorder, did not differ to nonanxious children on how threatening they viewed ambiguous (ER) stories, regardless of whether or not the stories included physical information. These findings add to a growing body of research that suggests that threat interpretation may not discriminate children with and without anxiety disorders (other than social anxiety), although it could not be tested within the current study whether this is consistent with the suggestion that ‘negative thoughts’ among clinically anxious children may better reflect reduced perceptions of control or coping rather than increased perceptions of threat (e.g., Alfano et al., 2002; Creswell et al., 2013; Waters et al., 2008).

Finally, it seems striking that no significant association between anxiety sensitivity and emotional reasoning was found. This may reflect some important differences between these constructs. The current findings, for example, suggest that children with social anxiety disorder may associate negative consequences with physical symptoms; however, there is no evidence that this leads them to change their interpretation of particular situations.

### 4.1 Limitations

This is the first study to investigate ER in children with a current anxiety disorder and therefore, to have confidence in the findings, replication is needed. In addition, the extension of these findings into adolescence warrants further research attention. The study is also not able to draw conclusions with regards to specificity in relation to other (non-social) anxiety disorders (e.g., panic disorder). Additionally, although all children in the socially anxious group met diagnostic criteria for social anxiety disorder, this was not always the primary disorder and the SA group had higher anxiety symptoms scores than the ANX group (even after items relating to social anxiety were excluded on the basis of parent report). It is therefore possible that the SA group scored higher on anxiety sensitivity and threat indices for the emotional reasoning stories than the ANX group because they had higher levels of anxiety symptoms. On the other hand, in diagnostic terms, children in the SA and ANX group had similar profiles on separation anxiety disorder, specific phobia, and generalized anxiety disorder (see Table 1). Consequently the only disorder that groups differed on was social anxiety disorder. Whether the findings reflect having social anxiety disorder specifically or are a function of increased anxiety severity (by virtue of having social anxiety disorder as well as the other anxiety diagnoses) requires investigation in future studies. It is also possible that the interview format with which the questionnaires were administered influenced responses from children (e.g., children might have underreported anxious responses). Finally, the cross-sectional nature of the study precludes any conclusions regarding the nature of the association between interpretation and anxiety in childhood, and prospective longitudinal and experimental work is required.

## 5 Conclusion

In summary, children aged 7–12 years, with social anxiety disorder, in comparison to children with other anxiety disorders and non-anxious children reported higher levels of AS and were more inclined to interpret ambiguous stories in a threatening way. These findings suggest that these cognitive constructs may be of particular relevance to the presentation and/or maintenance of social anxiety disorder. Further research is required to identify if a specific focus on these cognitive factors will improve treatment outcomes for social anxiety disorder.

## Role of funding source

Cathy Creswell was funded by MRC Clinician Scientist fellowship (G0601874). Anna Alkozei was funded by a University of Reading PhD Studentship.

## Conflict of interest

No conflicts of interest exist.

## Figures and Tables

**Table 1:** Child Diagnostic Characteristics.

Anxiety diagnoses	% Diagnosis		
	Social anxiety disorder *n=25*	Other anxious *n=25*	
Separation anxiety disorder	52	56	χ²(1) =.76
Social anxiety disorder	100	0	χ²(1)=43.95⁎⁎⁎
Specific phobia	48	40	χ²(1) =.32
Agoraphobia w/o panic disorder	4	8	χ²(1) =.35
Generalized anxiety disorder	68	44	χ²(1)=2.90
Obsessive-compulsive disorder	4	4	χ²(1)=1.00
Posttraumatic stress disorder	0	4	χ²(1) =.34
Anxiety disorder NOS	4	4	χ²(1)=1.00

	Comorbid diagnoses (%)	Other anxious	
	Social anxiety disorder			
	*n=25*	*n=25*	
Depressive disorder	16	4	χ²(1)=2.00
Externalizing disorder	36	20	χ²(1)=2.23

⁎⁎⁎ =*p*<.001

**Table 2:** Sample characteristics.

	Social anxiety disorder *N*=25	Other anxious *N*=25	Nonanxious *N*=25	
Age (months)	125.25	116.33	120.68	*F*(2, 57)=.79
(mean, SD)	(20.15)	(27.75)	(20.74)	
Gender (% female)	60%	60%	60%	*χ*²(1)=1.00
SCAS-c Total (mean, SD)	45.45 (16.99)^a^	36.60 (17.55)^b^	14.4 (11.69)^a^^,^^b^	*F*(2, 75)=25.07⁎⁎⁎
SCAS-c Social phobia (mean, SD)	8.15 (3.06)^a^	5.60 (4.22)^b^	2.40 (2.30)^a^^,^^b^	*F*(2, 75)=19.06⁎⁎⁎
SCAS-p Total (mean, SD)	39.9 (9.1)^a^^,^^c^	31.73 (10.47)^b^^,^^c^	13.56 (7.53)^a^^,^^b^	*F*(2, 75)=51.86⁎⁎⁎
SCAS-p Social phobia (mean, SD)	9.85 (3.73)^a^^,^^b^	5.06 (3.34)^b^	3.24 (2.14)^a^	*F*(2, 75)=26.74⁎⁎⁎
SCAS-c Corrected total (mean, SD)	37.35 (13.99)^a^	30.46 (16.08)^b^	11.28 (9.40)^a^^,^^b^	*F*(2, 57)=24.6⁎⁎⁎
SCAS-p Corrected total (mean, SD)	32.5 (7.54)^a^^,^^c^	23.66 (8.21)^b^^,^^c^	10.12 (5.58)^a^^,^^b^	F(2, 57)=58.61⁎⁎⁎

*Note.* SCAS-c: Spence Children's Anxiety Scale-child report; SCAS-p: Spence Children's Anxiety Scale-parent report; Corrected total: SCAS Total minus SCAS Social Phobia.

a denote groups that significantly differ on basis of posthoc analyses,

b denote groups that significantly differ on basis of posthoc analyses,

c denote groups that significantly differ on basis of posthoc analyses,

⁎⁎⁎ =*p*<.001.

**Table 3:** Childhood Anxiety Sensitivity Index (CASI).

	Social anxiety disorder *N*=25	Other anxious *N*=25	Nonanxious *N*=25	
CASI total (mean, SD)	20.24 (7.47)^a^^,^^b^	10.60 (7.65) a	7.24 (5.51)^b^	F(2, 69)= 23.71⁎⁎⁎
CASI embarrassment (mean, SD)	6.04 (2.15)^a^^,^^b^	4.04 (2.17) a	3.08 (2.25)^b^	F(2, 69)=11.71⁎⁎⁎
CASI additional anxiety (mean, SD)	10.80 (4.19)^a^^,^^b^	5.36 (4.83)^a^	3.24 (2.87)^b^	F(2, 69)=22.88⁎⁎⁎
CASI illness (mean, SD)	3.40 (2.41)^a^^,^^b^	1.20 (1.44)^a^	.92 (1.41)^b^	F(2, 69)=15.92⁎⁎⁎

*Note.* CASI: Childhood Anxiety Sensitivity Index.

a denote groups that significantly differ on basis of posthoc analyses.

b denote groups that significantly differ on basis of posthoc analyses.

⁎⁎⁎ =*p*<.001

**Table 4:** Emotional reasoning ambiguous stories with and without physical information.

	Social anxiety disorder *N*=25	Other anxious *N*=25	Nonanxious *N*=25	
Without physical information				
Fear threshold (mean, SD)	6.00 (2.21)^a^^,^^b^	10.16 (3.68)^a^	10.08 (3.45)^b^	F(2, 69)=16.53⁎⁎⁎
Fear frequency (mean, SD)	10.84 (2.33)^a^^,^^b^	6.69 (4.37)^a^	6.08 (3.22)^b^	F(2, 69)=17.87⁎⁎⁎
Fear ratings (mean, SD)	75.84 (27.93)^a^^,^^b^	34.04 (25.62)^a^	35.84 (29.54)^b^	F(2, 69)=24.11⁎⁎⁎
Negative feelings (mean, SD)	2.72 (.45)^a^^,^^b^	2.2 (.76)^a^	1.96 (.84)^b^	F(2, 69)=8.67⁎⁎⁎
Threat interpretation (mean, SD)	1.32 (1.10)^a^^,^^b^	.44 (.82)^a^	.24 (.43)^b^	F(2, 69)=12.33⁎⁎⁎

With physical information				
Fear threshold (mean, SD)	4.44 (1.47)^a^^,^^b^	8.44 (4.52)^a^	9.00 (3.42) b	F(2, 69)=14.00⁎⁎⁎
Fear frequency (mean, SD)	12.16 (2.17)^a^^,^^b^	7.72 (4.23)^a^	6.48 (3.64) b	F(2, 69)=21.26⁎⁎⁎
Fear ratings (mean, SD)	88.00 (29.63)^a^^,^^b^	45.84 (38.82)^a^	39.00 (32.30) b	F(2, 69)=20.76⁎⁎⁎
Negative feelings (mean, SD)	2.68 (.47)^a^^,^^b^	2.32 (.74)^a^	2.04 (.84) b	F(2, 69)=5.19⁎⁎⁎
Threat interpretation (mean, SD)	1.40 (1.00)^a^^,^^b^	.56 (.82)^a^	.40 (.64) b	F(2, 69)=10.26⁎⁎⁎

*Note*:

a denote groups that significantly differ on basis of posthoc analyses.

b denote groups that significantly differ on basis of posthoc analyses.

⁎⁎⁎ =*p*<.001.

## Footnotes

1 Muris et al. (2003) referred to these indices as ‘threat’ threshold and ratings. However, given that they refer to how ‘scary’ the child rates the story, this differs from common measures of ‘threat’ in which the child reports on what they think is going to happen, and we have relabeled this to distinguish it clearly from the measure of ‘threat’ interpretation.
